# Burden of cumulative risk factors associated with non-communicable diseases among adults in Uganda: evidence from a national baseline survey

**DOI:** 10.1186/s12939-016-0486-6

**Published:** 2016-12-01

**Authors:** Ronald Wesonga, David Guwatudde, Silver K. Bahendeka, Gerald Mutungi, Fabian Nabugoomu, James Muwonge

**Affiliations:** 1 School of Statistics and Planning, Makerere University, Kampala, Uganda; 2 School of Public Health, Makerere University, Kampala, Uganda; 3 Department of Internal Medicine St. Francis Hospital Nsambya, Kampala, Uganda; 4 Control of Non-communicable Diseases Desk, Ministry of Health, Kampala, Uganda; 5 Office of DVC, Kyambogo University, Kampala, Uganda; 6 Division of Socioeconomic Surveys, Uganda Bureau of Statistics, Kampala, Uganda; 7 East African Statistics Institute, Kampala, East Africa Uganda

**Keywords:** Non-communicable diseases, Cumulative risk factors, Disease burden, Prevalence, Uganda

## Abstract

**Background:**

Modification of known risk factors has been the most tested strategy for dealing with non-communicable diseases (NCDs). The cumulative number of NCD risk factors exhibited by an individual depicts a disease burden. However, understanding the risk factors associated with increased NCD burden has been constrained by scarcity of nationally representative data, especially in the developing countries and not well explored in the developed countries as well.

**Methods:**

Assessment of key risk factors for NCDs using population data drawn from 3987 participants in a nationally representative baseline survey in Uganda was made. Five key risk factors considered for the indicator variable included: high frequency of tobacco smoking, less than five servings of fruit and vegetables per day, low physical activity levels, high body mass index and raised blood pressure. We developed a composite indicator dependent variable with counts of number of risk factors associated with NCDs per participant. A statistical modeling framework was developed and a multinomial logistic regression model was fitted. The endogenous and exogenous predictors of NCD cumulative risk factors were assessed.

**Results:**

A novel model framework for cumulative number of NCD risk factors was developed. Most respondents, 38 · 6% exhibited one or two NCD risk factors each. Of the total sample, 56 · 4% had at least two risk factors whereas only 5.3% showed no risk factor at all. Body mass index, systolic blood pressure, diastolic blood pressure, consumption of fruit and vegetables, age, region, residence, type of residence and land tenure system were statistically significant predictors of number of NCD risk factors (*p* < 0 · 05). With exception to diastolic blood pressure, increase in age, body mass index, systolic blood pressure and reduction in daily fruit and vegetable servings were found to significantly increase the relative risks of exhibiting cumulative NCD risk factors. Compared to the urban residence status, the relative risk of living in a rural area significantly increased the risk of having 1 or 2 risk factors by a multiple of 1.55.

**Conclusions:**

The non-communicable disease burden is on the increase, with more participants reporting to have at least two risk factors. Our findings imply that, besides endogenous factors, exogenous factors such as region, residence status, land tenure system and behavioral characteristics have significant causal effects on the cumulative NCD risk factors. Subsequently, while developing interventions to combat cumulative risk factors of NCDs, the Ministry of Health needs to employ a more holistic approach to facilitate equitable health and sensitization across age, residence and regional divide.

## Background

World Health Organization (WHO), has recognized that it’s a right of every human being to live a healthy life [1]. However, based on the research evidence, a number of risk factors associated with non-communicable diseases (NCDs) jeopardize lives of millions of people world-wide [2, 3]. There are differences in risk factors exhibited per person depending on a number of distinguishing characteristics. The burden obviously increases cumulatively with the number of risk factors borne by an individual. This study considered only those risk factors [4] that were the main focus of the Uganda NCDs baseline population-based survey conducted in the year 2014. The five risk factors considered in the assessment were; daily smoking, consumption of less than five servings of fruit and vegetables per day, low physical activity levels, the high body mass index and raised blood pressure. In Uganda, prevalence of these risk factors has previously not been established nationally. However, some local area, small sample studies attempted to estimate the factors associated with NCDs [5–7]. Theoretically, the number of NCDs exhibited is a function of modifiable and non-modifiable factors [8–11] and is clearly assessed under data management section. Measurement of the burden of NCDs is a statistical problem whose solution lies in studies that evaluate the dynamics of NCD risk factors [12, 13]. Prevalence and incidences computed by WHO funded studies and others tend to estimate, although done scientifically, predicting the rates of occurrence and re-occurrence of NCD risk factors is yet another area of study [14, 15].

### Non-communicable diseases and their risk factors in Uganda

Uganda is one of the countries found in Sub-Saharan Africa where the burden of NCDs in terms of disability-adjusted life-years increases and continues to stress the already stressed and under-resourced health systems. The rising burden from NCDs, therefore, poses new challenges on the health system requiring more investments in sensitization, besides treatment.

Findings from the NCD risk factor baseline survey in Uganda reveal a high prevalence of hypertension at 26.5% among adults aged 18 to 64 years, with no significant differences between the four regions of Uganda [16]. Physical activity is a major risk of NCDs with 94.3% of participants in Uganda reported to meet the WHO threshold. Work-related physical activity of moderate intensity, and travel-related physical activity contribute most to the overall weekly duration, each contributing 49.6 and 25.2% respectively [17]. The overall prevalence of impaired fasting glucose in Uganda is estimated to be 2.0%, whereas that of diabetes mellitus is 1.4%. The prevalence of impaired fasting glucose is more (2.1%) among males than females (1.9%), whereas that of diabetes mellitus is 1.6 and 1.1%, respectively. The prevalence of both impaired fasting glucose and diabetes mellitus is comparatively low in Uganda, but significantly more among urban than rural dwellers; with most of the population not aware of their status [18]. Overall, the risk factors and disease burden posed by the NCDs are more prevalent in the elderly than the youth [19–22].

The main objective of this study was to assess the dynamics of factors associated with risk factors of NCDs in Uganda through establishing predictors for the number of risk factors exhibited by adults aged 18 to 69 years. The study also determined the prevalence for the number of key NCD risk factors exhibited. Subsequently, the probability of acquiring a certain level of risk factors was derived. This study augurs well with the WHO objective number six which focuses on monitoring trends and the social economic determinants of NCDs [23, 24].

## Methods

### Data sources

The study is based on analysis of data from a population based survey, which followed the WHO STEPwise approach to surveillance (STEPS) on risk factors for NCD [1, 25]. The survey had three parts. The first part of the survey (STEP 1) comprised of the collection of socio-demographic and behavioral information. This included the Global Physical Activity Questionnaire (GPAQ) for physical activity surveillance across countries [1, 26, 27]. The GPAQ was used to collect data on physical activity participation in three settings as well as on sedentary behavior, comprising of 16 questions. The domains included activity at work; travel to and from places and recreational activities. The second part of the survey (STEP 2) comprised of the collection of physical measurements such as height, weight, blood pressure, waist and hip circumference. The third part (STEP 3) comprised of the measurement of biochemical parameters to assess blood levels of total cholesterol (TC), High Density Lipoprotein cholesterol (HDL-C) and fasting blood glucose (FBG).

Participants in the survey were informed and their consent sought so as to get involved in the data collection for two consecutive days. First day was for providing responses to the survey questionnaire and taking of physical measurements of weight, height and waist circumference (STEP 1 and STEP 2), while the second day was used to measure fasting biochemical parameters using finger prick blood samples. All participants were requested to fast for at least 8 hours overnight for STEP 3 and not to indulge in exercise or smoking in the morning before data collection.

STEP 1 and 2 were carried out in the participant’s home. For STEP 3, participants were met at an agreed place in the enumeration area starting from 7:00 am. A blood sample from a finger prick for the measurement of TC, HDL-C and FBG was then taken and analysis done using the CardioChek® PA meter. Most of the participants had a very short distance to walk, and if substantial distance was envisaged, they came on a motorcycle or would be collected by the survey team vehicle. Participants eligible for blood sample collection were those who had an overnight 8-hour fasting with no exercise in the morning and did not smoke [28, 29].

### Data management

Five risk factors found to be associated with NCDs were used to generate the derived dependent variable (DDV). These were; tobacco smoking, less than five servings of fruit and vegetables per day, low physical activity levels, high body mass index and raised blood pressure. These were generated from an aggregation of related indicators for each of the five mentioned, thus, each of them represents a risk factor for the NCDs. For example, the indicator for smoking was derived from the questions; “Do you currently smoke any tobacco products, such as cigarettes, cigars, shisha or pipes?” and “Do you currently smoke tobacco products daily?” After data cleaning, dummy variables were generated for each. For the case, of smoking, it was ‘0’ when the response to the first question was ‘No’ and the response to the second question was ‘No’ otherwise coded as ‘1’ for “Yes”. Subsequently, row totals were generated for the five indicator variables to show the number of NCD risk factors per respondent.

The study employed a mixture of variables of different measurement scales for the independent variables. They include endogenous predictors, such as; systolic blood pressure, diastolic blood pressure and body mass index and the exogenous predictors such as; the number of daily fruit and vegetable servings consumed per day, location, residence, type of dwelling and land tenure system. Other personal predictors considered in the study included; age and sex of the respondent [7, 16, 25, 28, 30] as presented in Table 1. All variables underwent thorough data cleaning and verification before generating the risks and composite indicator. To avoid strong autocorrelations, correlation coefficients between the DDV and the predictors were generated and only those with correlation coefficients less than | ± 0 ⋅ 37| were considered for modeling. The study used the following definition for a risk factor as that portion or category of a variable whose continued co-existence or magnitude may endanger life of the host or subject. For example, low physical activity level is a risk factor, but physical activity itself is not; similarly, whereas high body mass index is a risk factor, body mass index itself is not. Thus the DDV was composited to a discrete numerical random variable that holds the counts per individual of the number of NCD risk factors.

**Table 1:** Description and measurement levels for the model variables

Number	Study variable	Variable definition	Measurement level
1	Risk factors for the derived dependent variable (DDV)	Daily tobacco smoking	Nominal
		Less than five servings of fruit and vegetables per day	Nominal
		Low physical activity levels	Nominal
		High body mass index	Nominal
		Raised blood pressure	Nominal
2	Endogenous observable factors (all had weak correlation coefficients with DDV of *r* < ± 0 ⋅ 37)	Age	Interval
		BMI	Ratio
		SBP	Interval
		DBP	Interval
		Servings	Interval
		Sex	Nominal
3	Exogenous observable factors	Region	Nominal
		Residence	Nominal
		Type of residence	Nominal
		Land tenure system	Nominal

### Statistical modeling

The multinomial logistic regression model, a statistical standard approach was employed to assess the dynamics of NCDs risk factors in Uganda, [31, 32]. This was applied conditional to the measurement level of modeling for the derived dependent variable (DDV) [33]. Under this modeling approach, the response variable was generated with three categories: 0=“No risk factor”, 1=“1 and 2 risk factors” and 2=“3 and more risk factors”. We modeled DDV as a polytomous response variable, to estimate the probability of occurrence of the different categories [32]. Cognizant of the fact that we were assessing determinants for occurrence of the categories of NCDs, rather than the levels of occurrence of the NCDs. Therefore, the multinomial logit model (MLM) was a natural choice, taking the form;

$$ p\left({Y}_i=k\right)=\frac{e^{\beta_k^{\hbox{'}}{X}_i}}{1+{\displaystyle {\sum}_{k=1}^{K-1}{e}^{\beta_k^{\hbox{'}}{X}_i}}} $$

The index, *i* is a set of predictors {1,2…} while k represents a set of categories for the response category, Y with the range belonging to the set {0,1,2} for the 3 categories of NCDs as classified. We chose k = 0 as a suitable base category against which the other categories are compared. The exponential beta coefficients thus represent the change in the odds of the response variable being in a particular category versus the reference category, associated with a one-unit change of the corresponding predictor variable. The usual shortcomings that arise with MLM relying upon the independence of irrelevant alternatives did not affect our model since we minimized chances of existence of irrelevant alternatives by exhausting the choices for the predictor variables.

## Results

### Distribution of number of key risk factors per person

The median number of risk factors per person was established as two risks with the number of NCD tending towards a positively skewed distribution. The proportion of the population exhibiting one or two risk factors was 38 · 6% each, while about 56 · 1% had at least two risk factors.

The distribution of exogenous predictors was studied. Table 2 shows details of their relationship with the number of NCD risk factors per person. The relationships of all the four categorical variables, region, residence, type of residence and land tenure system with the number of risk factors were statistically significant (*p* < 0.05). Overall, findings show that residents in the central region were more likely to have at least two NCD risk factors than any other region; followed by western, eastern and northern respectively. Central region hosts Kampala, the capital city of Uganda where most economic activities are carried out, hence making people more vulnerable to NCDs risk factors. Sensitization on the dangers of sedentary life style isn’t growing at the same rate. Similarly, urban residents were found to exhibit two or more risk factors than those in rural areas. Urban residence, like living in the central region is characteristic of urbanization that is impacting on the health of the population. Furthermore, differences in environmental factors and living conditions faced by the populations between rural and urban areas may explain differences in the burden of NCDs. Subsistence living conditions in rural areas have a toll on the inhabitants to work harder for their family’s survival. It was not surprising that respondents living in tenement type of residence registered more NCD risk factors than those living in independent residents. And similarly, respondents living in rented land tenure system had a higher significant association with two or more risk factors than those who own the land they lived on.

**Table 2:** Relationship between non-communicable disease risk factors and exogenous characteristics

Number of risk factors	0-risk factors (%)	1-risk factor (%)	2-risk factors (%)	3-risk factors (%)	4-risk factors (%)	5-risk factors (%)	Sample distribution (n)
Region							
Northern	5 · 8	43 · 4	38 · 3	10 · 6	1 · 8	0 · 0	707
Eastern	6 · 9	40 · 3	37 · 3	12 · 5	3 · 2	0 · 0	1,036
Central	3 · 9	35 · 8	39 · 4	17 · 3	3 · 5	0 · 1	1,359
Western	5 · 2	37 · 2	39 · 1	15 · 6	2 · 9	0 · 0	885
***χ*** ^2^ = 44 ⋅ 16*; p = 0.000*							
Residence							
Urban	3 · 7	33 · 0	39 · 9	18 · 9	4 · 5	0 · 0	1,084
Rural	5 · 9	40 · 7	38 · 1	12 · 8	2 · 5	0 · 0	2,903
***χ*** ^2^ = 51 ⋅ 74*; p = 0.000*							
Type of residence							
Independent housing	5 · 4	39 · 0	38 · 1	14 · 7	2 · 9	0 · 0	2,682
Tenement	2 · 1	31 · 7	45 · 0	17 · 3	3 · 9	0 · 0	716
Independent flat	0 · 0	47 · 8	30 · 4	21 · 7	0 · 0	0 · 0	23
Sharing	0 · 0	40 · 0	36 · 7	21 · 7	1 · 7	0 · 0	60
Boys Quarters	0 · 0	42 · 1	42 · 1	10 · 5	5 · 3	0 · 0	19
Garage	0 · 0	0 · 0	50 · 0	50 · 0	0 · 0	0 · 0	2
Hut	11 · 1	46 · 6	32 · 9	6 · 6	2 · 6	0 · 2	468
Other	0 · 0	38 · 5	15 · 4	38 · 5	7 · 7	0 · 0	13
***χ*** ^2^ = 136 ⋅ 22*; p = 0.000*							
Land Tenure System							
Owned	6 · 1	39 · 6	37 · 8	13 · 8	2 · 7	0 · 0	3,011
Rented	1 · 7	34 · 1	43 · 2	16 · 9	4 · 2	0 · 0	789
Supplied	1 · 8	40 · 4	31 · 6	21 · 1	5 · 3	0 · 0	57
Supplied	10 · 0	47 · 3	31 · 8	8 · 2	2 · 7	0 · 0	110
Other	13 · 3	20 · 0	46 · 7	20 · 0	0 · 0	0 · 0	15
***χ*** ^2^ = 63 ⋅ 99*; p = 0.000*							

The distributions of observable endogenous numerical predictors were studied. Table 3 shows the descriptive statistics and their relationship with number of NCD risk factors exhibited. An assessment of the relationship among the five numerical variables; age, body mass index, systolic blood pressure, diastolic blood pressure and fruit and vegetable servings per day is presented. As before, there is a consistent increase in the number of NCD risk factors with the increases in age, body mass index, systolic blood pressure, and diastolic blood pressure. The number of fruit and vegetable servings per day was found to have an inverse association with NCD risk factors exhibited by an individual; implying consumption of more fruits and vegetables reduces NCD risk factors.

**Table 3:** Distribution of NCDs by observable endogenous characteristics

Number of NCD risk factors		Age	Body mass index	Systolic blood pressure	Diastolic blood pressure	Fruit and vegetable servings
0 risk factors	n	211	211	211	211	208
	$\overline{x}$	33.71	20.95	118.42	77.95	8.58
	*s*	12.81	2.11	9.72	6.67	4.26
1 risk factor	n	1539	1499	154	1539	1531
	$\overline{x}$	33.25	21.39	117.69	76.73	2.65
	*s*	12.30	2.48	15.63	10.28	3.15
2 risk factors	n	1539	1363	1539	1539	1538
	$\overline{x}$	35.54	23.41	121.70	79.35	2.14
	*s*	12.85	6.51	29.23	18.40	2.27
3 risk factors	n	577	502	577	577	577
	$\overline{x}$	39.61	26.96	131.37	85.72	1.87
	*s*	13.69	14.60	34.66	22.52	1.64
4 risk factors	n	120	114	120	120	120
	$\overline{x}$	43.30	30.03	150.06	97.36	1.54
	*s*	13.51	24.29	27.80	18.93	1.15
5 risk factors	n	1	0	1	1	1
	$\overline{x}$	69.00	-	138.00	101.50	0.43
	*s*	-	-	-	-	-

Note: *n* sample size distribution, $\overline{x}$ average, *s* standard deviation, − implies no statistic to report

### Multivariate model for effects of key risk factors

To study, the dynamics of the multivariate effect of each of the factors on NCD risk factors, robust relative risk ratios were generated, by fitting a multinomial logit model (MLM) with results presented in Table 4.

**Table 4:** Determinants of cumulative risk factors associated with non-communicable diseases in Uganda

Reference category: 0 Risk Factors	1 or 2 Risk Factors		3 to 5 Risk Factors	
	RRR	*p*-value	RRR	*p*-value
Age	1 · 024	**0 · 000**	1 · 056	**0 · 000**
Body Mass Index	1 · 217	**0 · 000**	1 · 407	**0 · 000**
Systolic Blood Pressure	1 · 039	**0 · 000**	1 · 056	**0 · 000**
Diastolic Blood Pressure	1 · 008	0 · 348	1 · 070	**0 · 000**
Fruit vegetable servings	0 · 658	**0 · 000**	0 · 531	**0 · 000**
Region				
Northern^a^	1 · 000	-	1 · 000	-
Eastern	1 · 349	**0 · 000**	1 · 403	0 · 116
Central	1 · 501	0 · 240	1 · 858	**0 · 017**
Western	0 · 780	**0 · 044**	0 · 922	0 · 728
Residence				
Urban^a^	1 · 000	-	1 · 000	-
Rural	1 · 545	**0 · 000**	1 · 157	0 · 376
Sex				
Male^a^	1 · 000		1 · 000	
Female	0 · 851	0 · 697	0 · 836	0 · 636
Type of residence				
Independent house^a^	1 · 000	-	1 · 000	-
Tenement	0 · 855	0 · 698	0 · 863	0 · 089
Independent	1 · 800	**0 · 000**	1.700	**0 · 000**
Sharing house/flat/apartment	1 · 670	**0 · 000**	1.670	**0 · 000**
Boys Quarters	1 · 800	**0 · 000**	1.450	**0 · 000**
Garage	1 · 600	**0 · 000**	1.380	**0 · 000**
Hut	1 · 273	**0 · 000**	0 · 888	0 · 229
Other	1.268	**0 · 000**	1.560	**0 · 000**
Land Tenure System				
Owned^a^	1 · 000	-	1 · 000	-
Rented	3 · 739	0 · 091	4 · 196	0 · 066
Supplied free by employer	1 · 052	0 · 945	1 · 537	0 · 666
Supplied free or rent paid by relative or other person	0 · 683	**0 · 046**	0 · 759	**0 · 009**
Other	0 · 924	0 · 790	2 · 844	**0 · 000**
Constant	0 · 002	**0 · 000**	0 · 000	**0 · 000**

Note: *RRR* relative risk ratio, *DV* dependent variable, *NCD* non-communicable disease

Bold *p*-values indicate significant categories; ^a^a reference category with RRR = 1.000 and un-computed *p*-values; − implies no statistic to report

From the MLM, two models were derived; one for determinants of 1 or 2 risk factors and the other for determinants of 3 to 5 risk factors. The category 0 ‘=0 risk factors’ was used as the base or reference category. With exception of diastolic blood pressure, all the other endogenous observable factors, including; age, body mass index, systolic blood pressure, diastolic blood pressure and fruit and vegetable servings increased and had significant (*p* < 0 · 05) effects on the risk factors. Fruit and vegetable servings per day significantly reduce the NCD risk factors exhibited by factors of 0.658 and 0.531 for the 1 or 2 and 3 to 5 risk factors models respectively. Findings also showed that the constant that is, the effect on the mean number of NCD risk factors exhibited with no predictors was statistically significant.

The dynamic variations of exogenous observable factors were examined. Generally, the study established that living in the eastern region was a significant predictor of the first category (1 or 2 risk factors) of NCD risk factors exhibited, but not for the category 3 to 5 risk factors. Comparing the urban residents, living in a rural area significantly increased the risk of having 1 or 2 risk factors by a multiple of 1.545. For type of residence, the relative risk ratios were all positive. With independent-house as a reference category, living in a hut, garage, boys’ quarters or sharing significantly increased the chances of exhibiting 1 or 2 risk factors by over 1.3. Analysis of land tenure system revealed that for the land ‘supplied free or rent paid by relative or other person’ was a statistically significant predictor for NCD risk factors exhibited (*p* < 0 · 05) and could reduce the risk by a factor of 0.683 and 0.759 for the ‘1 or 2’ and ‘3 to 5’ risk factors respectively.

## Discussions

**Fig. 1:**
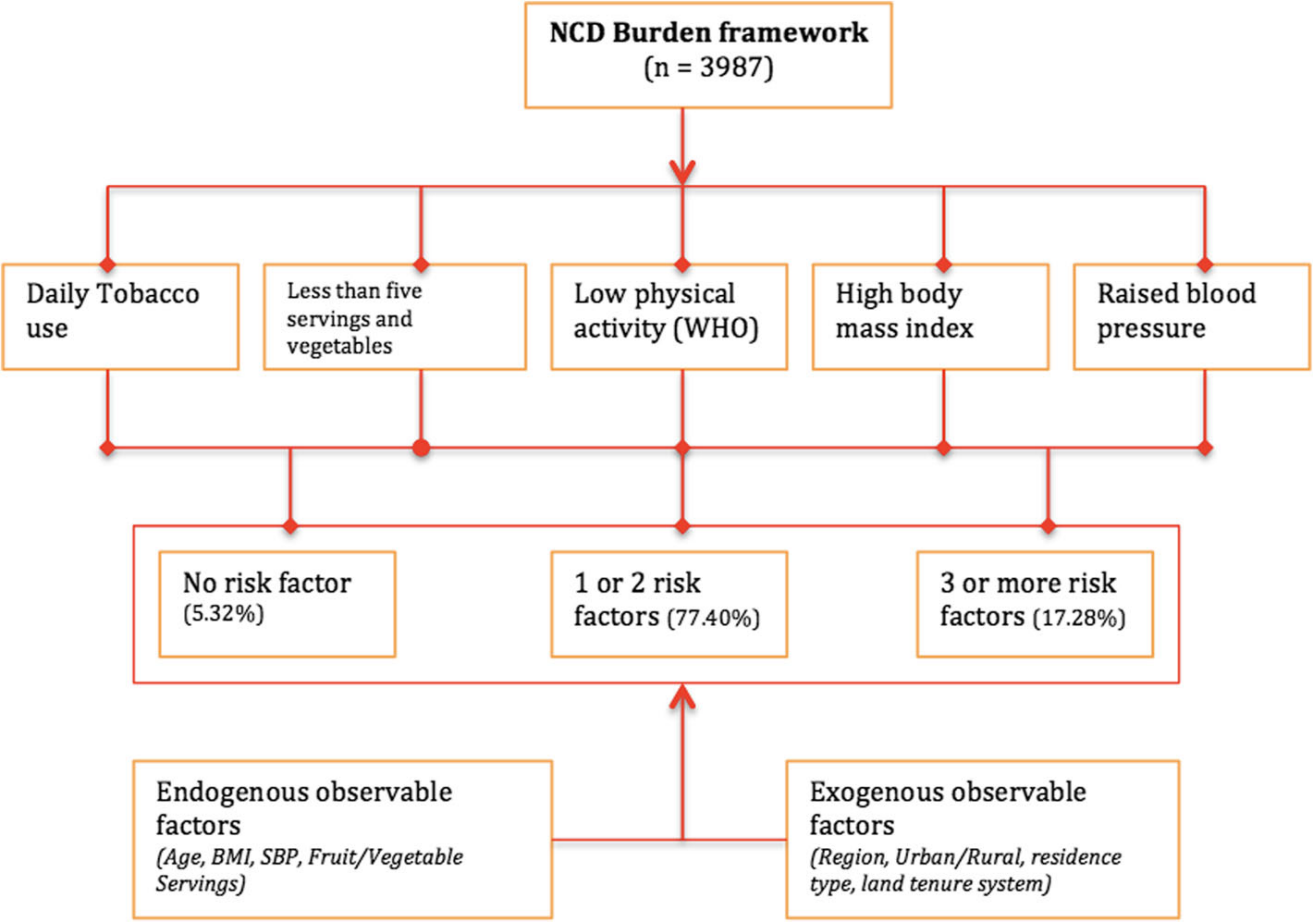
Framework for the burden of cumulative risk factors for non-communicable diseases

This study presents a novel framework that explores predictors of the risk factors associated with NCDs. The burden faced by individuals due to cumulative risk factors for NCDs was assessed including their prevalence rates and is demonstrated in Fig. 1.

This study defined a 5-category NCD risk factor to include; daily tobacco use, less than 5-daily servings of fruits and vegetables, low physical activity than WHO recommendation, high BMI and raised blood pressure. These variables were used to categorize and generate an index for number of risk factors. Further, three categories of risk factors were generated where a participant could present with none of them, one or two, and three or more risk factors. The significant proportion of the participants, 77% exhibited one or two of the risk factors (38.5% each) and over 90% exhibited at least 1 risk factor. These high proportions could have a significant influence on the productivity of the individual, increased burden on the healthcare system and eventually stifle economic development.

Evidently, as our findings show, NCD risk factors are on the increase and so are the variations in their associated factors relational and causal fcators [34, 35]. Relatedly, there are also variations in cumulative risk factors due to non-communicable diseases that directly affect the disease burden in families and communities. For instance, for two participants, while holding other factors constant, the one with two or more risks supposedly experiences greater disease burden than the one with one disease risk factor.

The effects of the risk factors derived from our model concur with predictors of the NCDs as noted in other studies [36–39]. For instance, there is a significant direct relationship between the number of NCD risk factors with body mass index, systolic blood pressure, but an inverse relationship with consumption of fruit and vegetables. Thus, higher BMI, systolic blood pressure and consumption of less than five servings of fruits and vegetables per day increases the relative risks of more cumulative NCD risk factors. A number of exogenous observable factors including: region, residence, type of residence and land tenure system significantly determine the cumulative number of risk factors for the NCDs. Participants residing in the urban areas were more likely to have two or more risk factors than those in rural areas. Related studies concur that urban residence is a primary risk factor for NCDs impacting on the health of the population [20, 40, 41]. Regionally, in Uganda, urbanization is more realized in the central region, and this, too was significantly associated with number of NCD risk factors. Differences in exogenous observable factors and living conditions of the population between rural and urban areas explain differences in the burden of NCDs. Subsistence living conditions in rural areas have a toll on the inhabitants forcing them to work sometimes under very difficult conditions for their family’s survival. Participants living in a hut had less likelihood of increased NCD risk factors. A hut in the context of Uganda is a proxy indicator for poverty, which measures the level of difficulty of earning a living. Given that over 90% of the participants met the WHO physical activity requirement, this factor was dropped during the analysis phase [17]. Findings about land tenure system reveal that persons living on land supplied free by the employer bore reduced chances of having multiple NCD risk factors. Land in Uganda is mainly under private ownership, making it very expensive for individuals to acquire. However, given that it is not sustainable for every employer or otherwise to give free land to employees, government needs to regulate the cost of land so as it is more affordable.

## Conclusions

In this study, we assessed determinants for cumulative risk factors of an individual participant associated with NCDs and subsequent burden this causes. Findings show that besides the endogenous factors such as; body mass index, blood pressure and consumption of fruit and vegetables, there were significant effects on the NCD risk factors among participants predisposed to exogenous factors such as region, residence status and land tenure system. The study showed that risk factors associated with NCDs are on the increase resulting in the burden due of cumulative risk factors presented by an individual. This burden due to risk factors of the NCDs manifests itself in many ways including; late presentation due to the inability of the majority to go for regular tests mainly because they cannot afford the costs of testing and treatment itself. As a consequence of late presentation arises the multiple patient-complications such as blindness, kidney diseases, stroke and heart attacks. With the compounding high poverty rates, the ultimate burden results in high rates of morbidity and mortality.

Notwithstanding, the study was affected by sampling error and some biases in the data due to refusals, especially when it came to testing for diabetes and the requirements such as fasting before blood glucose, measurements could be obtained accurately. Consequently, risk factors of NCDs were restricted to a limited set of predictors.

Despite those limitations, findings of this study provide an indication, both in magnitude and direction of the burden of cumulative risk factors to the general population and specifically to persons living with NCD (s) and the distribution of NCD burden nationally. Therefore in developing interventions to combat NCD prevalence, the government of Uganda needs to employ a more holistic approach to develop and operationalize NCD policy. The policy should provide measurable indicators for each NCD and associated risk factors; a strategy for prevention of harmful use of alcohol and a ban on consumption of unrecorded alcohol and regular national NCD health education. Guidelines, such as NCD screening, diet and physical activity and IEC materials on NCD prevention and control should be developed and translated in local languages. Specifically, the Government of Uganda should reduce the equity gap between rural and urban residents through actions geared to service delivery towards on social, economic and environmental welfare. The Ministry of Health (MOH) should elevate the NCD Desk into a Directorate with a reasonable budget. This could help to implement NCD preventive measures so as to promote equity to access health services including sensitization drives that increase awareness and change attitudes to lifestyle factors.

This study was the first to assess the burden imposed by the cumulative risk factors associated with NCDs. We recommend a study on causal relationships between potential risk factors and NCDs. The Ministry of Health in Uganda, in collaboration with statistical research organizations should institute a more comprehensive national survey to determine the interplay of socio-economic, environmental and cultural factors on the cumulative number of risk factors associated with the Non-Communicable Diseases.

## Abbreviations

BMI: Body mass index
DDV: Derived dependent variable
FBG: Fasting blood glucose
HDL-C: High-density lipoprotein cholesterol
IEC: Information Education and Communication
MLM: Multinomial logistic model
MOH: Ministry of health
NCDs: Non-communicable diseases
TC: Total cholesterol
UNCST: Uganda national council for science and technology
WHO: World Health Organization
